# Idiopathic Pulmonary Fibrosis and Lung Cancer: Mechanisms and Molecular Targets

**DOI:** 10.3390/ijms20030593

**Published:** 2019-01-30

**Authors:** Beatriz Ballester, Javier Milara, Julio Cortijo

**Affiliations:** 1 Department of Pharmacology, Faculty of Medicine, University of Valencia, 46010 Valencia, Spain; julio.cortijo@uv.es; 2 CIBERES, Health Institute Carlos III, 28029 Valencia, Spain; xmilara@hotmail.com; 3 Pharmacy Unit, University Clinic Hospital of Valencia, 46010 Valencia, Spain; 4 Institute of Health Research-INCLIVA, 46010 Valencia, Spain; 5 Research and teaching Unit, University General Hospital Consortium, 46014 Valencia, Spain

**Keywords:** idiopathic pulmonary fibrosis (IPF), lung cancer (LC), non-small cell lung cancer (NSCLC)

## Abstract

Idiopathic pulmonary fibrosis (IPF) is the most common idiopathic interstitial pulmonary disease with a median survival of 2–4 years after diagnosis. A significant number of IPF patients have risk factors, such as a history of smoking or concomitant emphysema, both of which can predispose the patient to lung cancer (LC) (mostly non-small cell lung cancer (NSCLC)). In fact, IPF itself increases the risk of LC development by 7% to 20%. In this regard, there are multiple common genetic, molecular, and cellular processes that connect lung fibrosis with LC, such as myofibroblast/mesenchymal transition, myofibroblast activation and uncontrolled proliferation, endoplasmic reticulum stress, alterations of growth factors expression, oxidative stress, and large genetic and epigenetic variations that can predispose the patient to develop IPF and LC. The current approved IPF therapies, pirfenidone and nintedanib, are also active in LC. In fact, nintedanib is approved as a second line treatment in NSCLC, and pirfenidone has shown anti-neoplastic effects in preclinical studies. In this review, we focus on the current knowledge on the mechanisms implicated in the development of LC in patients with IPF as well as in current IPF and LC-IPF candidate therapies based on novel molecular advances.

## 1. Introduction

Idiopathic pulmonary fibrosis (IPF) is a form of chronic, progressive fibrosing interstitial pneumonia of unknown cause that occurs primarily in older adults. IPF is associated with the histopathologic and/or radiologic pattern of usual interstitial pneumonia (UIP) that is limited to the lungs [1]. The disease course of IPF is variable and somewhat unpredictable, nevertheless, progression to end-stage respiratory insufficiency and death after the onset of symptoms from diagnosis is 2–4 years [2]. Pulmonary and extrapulmonary comorbid conditions, such as lung cancer (LC), are commonly associated to IPF, altering the disease course and mortality. Links between pulmonary fibrosis and LC have been suggested as early as 1965 [3] and are based on the multiple common genetic, molecular, and cellular processes that connect both diseases and can predispose the patient to develop IPF and LC.

On its own, pulmonary fibrosis is a risk factor for developing lung carcinogenesis [4,5,6,7]. Moreover, elder age, male sex, history of smoking, and coexisting emphysema are also strong risk factors that contribute to developing LC in IPF patients [8,9,10,11,12,13,14,15,16,17,18,19]. The prevalence of LC in IPF patients ranges from 2.7% to 48% [8,9,13,14,15,16,17,18,19,20] (Table 1) and is significantly higher than in the general population [21]. Otherwise, the incidence of LC in IPF patients is reported to be 11.2–36 cases per 1,000 persons per year [8,20,22], which increases with each year following IPF diagnosis [15,18]. Moreover, IPF patients that are diagnosed with LC have a reduced mean survival time (1.6–1.7 years), compared to IPF patients without LC diagnosis [10,17] and Kato et al. reported 53.5%, 78.6%, and 92.9% as 1-, 3-, and 5-year all-cause mortality rates after LC diagnosis in IPF patients [8].

## 2. Histological Subtypes and Parenchymal Distribution of Lung Cancer in Idiopathic Pulmonary Fibrosis

In the general population, the predominant type of LC is non-small cell lung cancer (NSCLC). Likewise, NSCLC is the predominant type of LC in LC-IPF patients. Furthermore, adenocarcinoma (ADC) is the most common subtype of histological NSCLC in the general population [23]. However, the most frequent histological subtype of LC in IPF has been controversial over the past few years (Table 2). Recently, the majority of studies have shown that squamous cell carcinoma (SQC) is the most frequent type of LC in IPF patients, while ADC is the second most frequent [8,9,10,14,15,17,18,19,24,25,26,27,28,29]. Moreover, some isolated cases of large cell carcinoma and small cell lung cancer (SCLC) have also been reported [8,14,18,27].

Similarly to fibrotic lesions, lung carcinomas are generally more frequently found in the peripheral area of the lungs in IPF patients, i.e., in the lower lobes [3,8,16,25,30,31,32], and are associated with honeycomb lesions, developing from honeycomb areas or in the border between honeycombing and non-fibrotic areas, and epithelial metaplasia [33,34,35]. In general, squamous metaplasia, but not cuboidal cell metaplasia or bronchial cell metaplasia, have been observed more frequently in LC-IPF patients than in IPF patients without lung carcinoma. Then, it is speculated that it might reflect a constitutional susceptibility of IPF patients of developing lung carcinoma [36]. 

## 3. Cell Types and Cellular Processes Involved in Lung Cancer Associated with Pulmonary Fibrosis

### 3.1. Cell Transformations in the Mesenchymal Phenotype

**Figure 1:**
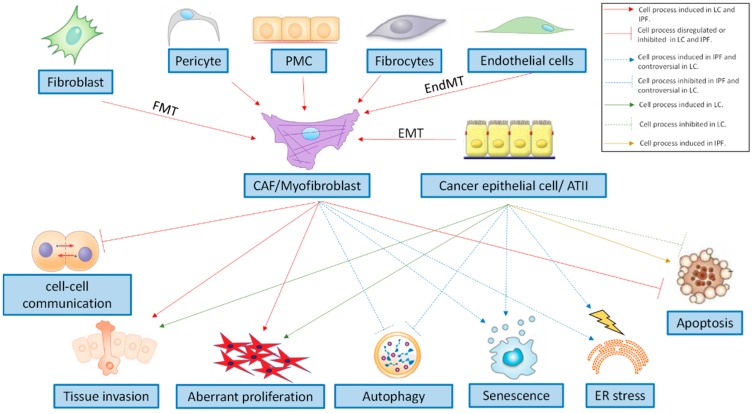
Cell types and cellular processes involved in lung cancer (LC) and idiopathic pulmonary fibrosis (IPF). Lung resident fibroblasts, pericytes, pleural mesothelial cells (PMC), circulating fibrocytes, vascular endothelial cells, and epithelial cells (Alveolar type II cells (ATII) in IPF and cancer epithelial cells in LC) are transformed to IPF myofibroblast or mesenchymal phenotype and cancer-associated fibroblasts (CAFs). Myofibroblasts and cancer cells are characterized by altered cell-cell communication, migration properties, and tissue invasion through basement membranes and the extracellular matrix. IPF myofibroblasts and ATII cells acquire senescent identities, but the presence of this phenotype is controversial in LC. Otherwise, endoplasmic reticulum (ER) stress is induced in IPF, while autophagy is defective in IPF. However, the function of both processes is controversial in LC. Finally, apoptosis is induced in ATII cells, but IPF myofibroblasts and cancer cells evade apoptosis.

IPF is characterised by an excess of myofibroblasts that are persistently activated in fibrotic lungs [37]. The activated myofibroblasts are stellate- or spindle-shaped, and are characterised by the secretion of extracellular matrix (ECM) components (a characteristic shared with fibroblasts), such as collagen type I, and by the formation of contractile apparatus (a characteristic shared with airway smooth muscle cells), such as α-smooth muscle actin (α-SMA) microfilaments [38]. In IPF lungs, myofibroblasts have heterogeneous phenotypes [39] (Figure 1). The classic concept is that tissue injury induces the activation of resident fibroblasts to proliferate and express constituents of the ECM and α-SMA fibres [40]. One contemporary theory is that tissue injury in the presence of transforming growth factor (TGF-β) induces epithelial to mesenchymal cell transition (EMT) [41,42]. In detail, EMT is part of an unabated form of wound healing in which alveolar type II (ATII) cells [41,42,43] can serve as a source for the increased myofibroblast-like pool that eventually leads to organ destruction if the primary inflammatory insult that triggered the wound healing is not removed or attenuated [44]. Another contemporary theory of myofibroblast activation is that circulating fibrocytes that originate from the bone marrow are mesenchymal progenitor cells that home and extravasate into sites of tissue injury, and differentiate into the myofibroblast-like phenotype [45,46] in response to TGF-β and endothelin 1 [47,48,49]. Pulmonary arterial endothelial cell to mesenchymal transition (EnMT) has been suggested as another source of myofibroblasts that potentially contribute to lung fibrosis and pulmonary hypertension and is often associated with IPF, which portends a poor prognosis [50,51]. Finally, there are two emerging theories that consider pleural mesothelial cells or lung pericytes as significant sources of lung myofibroblasts in IPF [52,53,54,55,56]. 

Similarly to IPF, cancer-associated fibroblasts (CAFs) are also important players in LC because they exhibit mesenchymal-like features and have heterogeneous phenotypes (Figure 1) [57]. Lung resident fibroblasts surrounding the malignancy are thought to be the first responders to the site of insult that the tumour creates [58]. Resident epithelial cells—generally bronchiolar epithelial cells—can also undergo a partial and possibly reversible EMT during the early steps of carcinogenesis, cancer invasion, and metastasis [44,59]. Likewise, fibrocytes recruited from the peripheral circulation have also been suggested as a potential source of CAFs [58,60,61]. It has also been observed that up to 40% of CAFs arise as a consequence of EndMT in two different murine cancer models, suggesting that EndMT may play a role in tumour angiogenic sprouting into adjacent tissue [62]. In the tumour environment, vascular pericytes have also been associated with tumour vasculature. Pericytes that detach from the tumour microvasculature have been shown to undergo differentiation into stromal fibroblasts via the action of platelet-derived growth factor-BB (PDGF-BB), which significantly contributes to tumour invasion and metastasis [63]. Finally, pleural mesothelial cells are also hypothesised as a source of CAFs, where a mesothelial precursor lineage has been identified as being capable of clonally generating fibroblasts in the lungs, kidney, liver, and gut [64]. Also, it has been reported that an overexpression of mesothelin in lung ADC is induced by tobacco-related carcinogens [57].

### 3.2. Common Cellular Processes in Lung Cancer Associated with Pulmonary Fibrosis

#### 3.2.1. Apoptosis and Autophagy

Growing evidence indicates a prominent role of enhanced endoplasmic reticulum (ER) stress in IPF, resulting in an unfolded protein response (UPR) [65,66]. This response mechanism activates biochemical pathways to meet the demands of protein folding. However, if that is no longer feasible, a terminal UPR directs alveolar epithelium cells towards apoptosis. By contrast, IPF myofibroblasts and cancer cells escape apoptosis [67,68]. Regarding the role of ER-stress in tumourigenesis, it is controversial [69,70], nevertheless, recent evidence shows that ER stress may attenuate senescence and promote tumorigenesis [71] (Figure 1). 

Despite elevated ER stress, there is evidence that autophagy is defective in IPF [72,73,74] (Figure 1), which promotes lung fibroblast differentiation into myofibroblasts via excessive ECM production [72,74,75] and fibroblast resistance to apoptosis [76]. In LC, autophagy functions as a double-edged sword because it suppresses tumorigenesis in a limited number of contexts while facilitating it in most others [77]. In fact, it has been observed that autophagy can promote or inhibit apoptosis under different cellular contexts within the same tumour cell population. Therefore, therapeutic targeting of autophagy in cancer is sometimes viewed as controversial [78].

#### 3.2.2. Cellular Proliferation

Evidence strongly supports the persistent activation of proliferative signalling pathways in IPF (Figure 1). In fact, myofibroblasts sustain their own growth through the autocrine production of TGFβ1 and partly lose their ability to produce anti-fibrotic prostaglandin E2 (PGE2) [79]. Further, they show a lack of response to the inhibitory activity of PGE2 [80], and to other antiproliferative signals [81]. The receptors for platelet-derived growth factor (PDGF), vascular endothelial growth factor (VEGF), and fibroblast growth factor (FGF) have also recently been implicated in the sustained proliferation signalling of pulmonary fibroblasts [82]. However, this persistent activation is not definitively linked with aberrant fibroblast proliferation in vivo [83,84,85] and the role of excessive fibroblast proliferation as a pathogenic mechanism of IPF is unclear. By contrast, aberrant proliferation of cancer cells and sustained proliferative signalling has been described as “arguably the most fundamental trait of cancer cells” [68]. 

#### 3.2.3. Altered Cell-Cell Communications

Intercellular channels that are formed by connexins are essential for the synchronisation of cell proliferation and tissue repair [86]. In particular, the expression of connexin 43 (Cx43) is considered crucial in fibroblast-to-fibroblast communication. In IPF fibroblasts, Cx43 expression is strongly down-regulated, leading to a loss of proliferative control [87]. Similarly, cancer cell lines from mouse and human lung carcinoma have low levels or an absence of Cx43 expression [88], which results in reduced cell-to-cell communication (Figure 1); this may explain both the release of cells from contact-inhibition control, and the uncontrolled proliferation that characterises this disease. 

#### 3.2.4. Senescence

Analyses of cell types in the lungs of both human IPF and the bleomycin-injured mouse model have demonstrated that fibroblasts and epithelial cells acquire senescent identities [89]. Senescence appears to be a central phenotype that promotes lung fibrosis through increased production of a complex senescence-associated secretory phenotype (SASP) based on growth factors, cytokines, chemokines, and matrix metalloproteinases, as well as acquired apoptosis resistance in IPF fibroblasts [90] (Figure 1). However, therapeutic management of cell senescence is controversial in cancer. On the one hand, cell senescence could limit the replicative capacity of cells and ultimately prevent their proliferation in different stages of malignancy, while providing a protective barrier to neoplastic expansion [91]. On the other hand, it has been proposed that senescent fibroblasts may promote tumour progression, possibly by secreting an SASP based on certain matrix metalloproteases, growth factors, and cytokines [92].

#### 3.2.5. Tissue Invasion

IPF lung fibroblasts are characterised by their ability to invade through the basement membrane and the ECM via the action of metalloproteinases [93,94]. This characteristic is also an important hallmark of cancer (Figure 1). Unlike cancers that can disseminate over long distances because they acquire further invasive mechanisms, fibrotic lung fibroblasts are restricted to local invasion [95]. The capacity of cancer cells to infiltrate the surrounding tissue is strictly related to the expression of laminin, heat shock protein 27, and fascin [96,97,98]. Interestingly, in IPF, it has been shown that bronchiolar basal cells surrounding the fibroblast foci express large amounts of these proteins, which induce cell motility and invasiveness of myofibroblasts [99]. Therefore, targeting these molecules may be a feasible strategy to restrain myofibroblast tissue invasion in LC-IPF patients.

#### 3.2.6. Inflammation

The role of inflammation in IPF is controversial, although evidence shows the existence of a predominant phenotype of fibrosis-associated macrophages (FAMs) that are alternatively activated. These are an M2 phenotype of FAMs [100] that facilitate the enhanced production of FGFs [101], profibrotic cytokines [102,103], and matrix metalloproteinases [104]. Like FAMs, tumour-associated macrophages also display an M2 phenotype and support tumour growth through their ability to promote angiogenesis, activate mesenchymal cells, remodel the matrix, and suppress effector T-cell responses [105,106]. Thus, M2 macrophages could be considered key effectors in the development of LC associated with pulmonary fibrosis.

## 4. Principal Fibrogenic Molecules and Signal Transduction Pathways Participating in Lung Cancer Associated with Pulmonary Fibrosis

### 4.1. Growth Factors

TGFβ is a major profibrotic growth factor and is often chronically overexpressed in cancer and fibrosis (Table 3) [107]. TGFβ can be activated by αVβ6 integrin and, in IPF, TGFβ1 mediates fibrogenesis by antiproliferative action and apoptosis in alveolar epithelial cells (AECs) or by stimulation of fibroblast differentiation to myofibroblasts, synthesis of ECM proteins, and inhibition of ECM degradation [108,109]. TGFβ also induces the production of fibrogenic or angiogenic growth factors and is known to strongly elicit EMT and EndMT. In the early stages of cancer pathogenesis, TGFβ acts as a tumour suppressor because it inhibits the growth of many cell types and delays the appearance of primary tumors. However, after the appearance of them, TGFβ promotes tumour progression, because it can induce EMT and EndMT, and suppresses immune surveillance. Therefore, during tumour progression, TGFβ triggers the formation of spontaneous lung metastases. Finally, TGFβ is also central in the development of the tumour stroma because TGFβ1 also activates CAFs [107,110].

Tyrosine kinase receptor ligands, such as PDGF, VEGF, and FGF, are aberrantly expressed in LC and IPF (Table 3) [82]. In IPF, PDGF plays an important role in inducing the secretion of ECM components and growth factors in fibroblasts [111]. It also promotes fibroblast proliferation and recruits fibrocytes to the lung [112,113]. Furthermore, TGFβ1, FGF, and tumour necrosis factor-α exhibit PDGF-dependent profibrotic activity [114,115]. Otherwise, PDGF signalling is also important for tumour growth, angiogenesis, and lymphangiogenesis in cancer [113]. In fact, it has been shown that crenolanib (PDGF receptor inhibitor) is capable of suppressing proliferation and inducing apoptosis in a dose-dependent manner using A549 cells as a NSCLC model system. Moreover, it has been shown that crenolanib-treated A549 cells have reduced migratory activity in response to inducers of chemotaxis, and the antitumor activity of this drug has been confirmed in an NSCLC xenograft tumor model [116]. Finally, it has been observed that PDGF regulates VEGF expression in NSCLC via an autocrine mechanism [117], and is also involved in the recruitment of CAFs to the tumour mass [113]. The contribution of VEGF to IPF is not fully understood because there is still debate on the role of vascular remodelling in IPF [118]. However, in addition to the role of VEGF in tumour angiogenesis, accumulating evidence suggests that it can act directly on cancer cells to regulate growth, migration, and production of several pro-angiogenic factors [119]. FGF is also released by damaged epithelial cells and activated fibroblasts during the remodelling processes [120,121]. It was found that FGF signalling is required for fibroblast expansion within fibrotic areas [122]. FGF can also affect the proliferation, treatment sensitivity, and apoptosis of LC cells [123]. 

Another important fibrogenic growth factor in LC is connective tissue growth factor (CTGF). In IPF, CTGF induces fibroblast proliferation and ECM deposition [124]. By contrast, it has been observed that CTGF inhibits metastasis and invasion of human lung ADC [125], and its expression is suppressed in many NSCLCs [126] (Table 3). 

Several other growth factors are involved in IPF and LC [127,128,129], but we have not included them in this review because they are not the focus of the new therapies currently being developed. 

### 4.2. Lysophosphatidic Acid (LPA)

LPA, a profibrotic mediator with proinflammatory activity, is released by platelets during epithelial injury [130]. Extracellular production of LPA is catalysed by autotaxin (ATX) and further regulated by phospholipid phosphatases (PLPP). IPF patients have increased LPA levels [131] in their bronchoalveolar lavage fluid (BALF) (Table 3), and recently, it has been shown that LPA signalling mediates both the fibroblast recruitment and vascular leakage induced by lung injury in a bleomycin model of pulmonary fibrosis [131]. Moreover, it has recently been shown that the ATX/PLPP3-LPA/LPA receptor 1 (LPAR1) axis has a procarcinogenic role in lung carcinogenesis [132].

### 4.3. Cytokines and Chemokines

Epithelial injury causes an imbalance in T-helper type 1 (Th1)/type 2 (Th2) cytokine expression, which results in a stronger Th2 response. In particular, there is evidence that interleukin 13 (IL-13) [133] plays a dominant role in the pathogenesis of fibrosis in the lungs of IPF patients (Table 3) [134]. IL-13 triggers the transformation of fibroblasts to myofibroblasts via the TGFβ-dependent and -independent pathways, while also inducing epithelial apoptosis [135,136,137]. Similarly to IPF, a pattern of Th2 cytokine expression has also been identified in NSCLC [138]. With respect to IL-13, a recent study observed the highest expression level of IL-13 in LC in the SQC subtype, followed by the ADC subtype [139]. Moreover, a clear association between IL-13 receptor subunit alpha-2 overexpression and poor survival in resected NSCLC patients has been shown [140]. There are other profibrotic cytokines besides IL-13 that are also associated with IPF. For example, chemokine ligand 2 (CCL2) has been reported to be present in the BALF of IPF patients at significant concentrations [141] (Table 3). In IPF, CCL2 has been shown to induce the differentiation of developing T-cells into type 2 cells [142], and to stimulate collagen synthesis and TGFβ expression in lung fibroblasts [141]. In LC, the CCL2/CCR2 (chemokine receptor type 2) axis is also important in several aspects of tumorigenesis. One of its most important roles is the generation of new vascular structures that allow tumour growth [140]. However, there is evidence of an association between CCL2 in cancer cells and better survival in NSCLC patients [143]. 

### 4.4. Reactive Oxygen Species (ROS)

ROS production by ATII cells results in oxidative stress, which induces apoptosis of epithelial cells, activates intracellular signalling pathways, and upregulates the synthesis of profibrotic cytokines that ultimately leads to tissue injury and fibrosis [144]. In this context, upregulation of NADPH oxidase 4 (NOX4) has been reported in pulmonary fibroblasts and other relevant cells in IPF. In the same way, NOX4 has been reported to be overexpressed in NSCLC (Table 3), contributing to cell proliferation and metastasis [145]. Furthermore, following ROS overproduction, alterations in DNA methylation patterns and specific histone modifications lead to aberrant gene expression, and possibly trigger the multistage process of carcinogenesis [144]. In addition, antioxidant molecules that mitigate oxidative stress, such as Nrf2, have also been reported to be dysregulated in both diseases [146,147] (Table 3). As such, they are proposed to be future targets for anti-IPF/LC treatment.

### 4.5. Mucins

Significant overexpression of the secreted Mucin 5B (Muc5B) protein has been found in IPF lungs (Table 3) and it is hypothesised that excess Muc5B impairs the mucosal host defence; in turn, this may interfere with alveolar repair and leads to the development of idiopathic interstitial pneumonia [148]. In this context, *MUC5B* expression has been associated with a high risk of distant metastasis in NSCLC patients and poorer prognosis in ADC patients [149,150]. 

We have also observed IPF overexpression of the transmembrane mucins, Muc1 [151], Muc4 [152], and Muc16 (unpublished data), which may be involved in the molecular processes that lead to the development of pulmonary fibrosis [151,152,153]. In addition, the extracellular region of Muc1 contains the KL-6 epitope, which is proposed to be a useful biomarker for evaluating disease activity and predicting clinical outcomes in IPF [154]. Similarly, these transmembrane mucins have previously been considered clinically relevant proteins that are aberrantly overexpressed in lung carcinogenesis [155]. In fact, Muc1 is a target in several preclinical and clinical trials for cancer treatment [156,157]. Concurrently, there is evidence that galectin 3 is a promising target for IPF [158] because it has a profibrotic action [159] that is partly mediated by binding to Muc1 [160]. Recently, the potential of galectin-3 as a therapeutic target in cancer has been highlighted since it is capable of modulating anti-tumour immunity [161].

### 4.6. Embryological Pathways

There is also evidence that some embryological pathways are reactivated or deregulated in fibrotic diseases (Table 3) [162]. For example, the Wnt/β-catenin pathway is overexpressed in the lung tissue of IPF [163] and LC patients [164]. This pathway regulates the expression of molecules involved in tissue invasion, such as matrilysin, laminin, and cyclin-D1, which induces the EMT process. Most importantly, this pathway is involved in biologically relevant cross talk with TGF-β [163]. 

The Sonic hedgehog (shh) pathway is also aberrantly activated in IPF, mainly in epithelial cells that line honeycomb cysts [165,166]. The overexpression of the shh pathway promotes increased susceptibility to epithelial cell apoptosis and increased resistance to fibroblast apoptosis [167]. This pathway is also reactivated at the early stage of oncogenesis by cancer stem cells and leads to paracrine action on other tumour cells, resulting in tumour growth, tumour spread, and EMT. In LC, reactivation of the shh pathway is involved in the development of resistance to all the main treatments of LC [168]. 

Finally, the Notch signalling pathway is also reactivated in AECs, induces α-SMA expression in fibroblasts, and mediates EMT in AECs [52]. In the same way, abnormal expression of the members of the Notch signalling pathway is a relatively frequent event in patients with NSCLC [169,170]. It has been demonstrated that members of the Notch signalling pathway may be potential biomarkers for predicting the progression and prognosis of patients with NSCLC. Furthermore, Notch signalling promotes the proliferation of NSCLC cells or inhibits apoptosis of NSCLC cells [171].

### 4.7. PI3K/AKT/mTOR Pathway

The phosphoinositide 3-kinase (PI3K)/protein kinase B (AKT)/mammalian target of rapamycin (mTOR)-dependent pathway is dysregulated in fibroproliferative diseases, like pulmonary fibrosis (Table 3) [172]. In fact, overexpression of class I isoform p110γ in lung homogenates occurs in IPF patients [173], and has been shown to activate the downstream signalling of several key profibrotic growth factors implicated in IPF, including PDGF and TGFβ1 [174,175], as well as abnormal proliferation of epithelial basal cells [173] and TGF-β-induced fibroblast proliferation and differentiation [176]. Moreover, it has been observed that the suppression of phosphatase and tensin homologue mediates matrix-mediated resistance to apoptosis [174]. Phosphatase and tensin homologue are negative regulators of PI3K that in turn activate AKT. De-regulation of the PI3K/AKT/mTOR pathway is also involved in NSCLC and has been associated with high grade tumours and advanced disease. Furthermore, abnormalities in this pathway are more common in SQC than in ADC of the lung [177].

## 5. Genetic and Epigenetic Alterations in Lung Cancer Associated with Pulmonary Fibrosis

### 5.1. Genetic Alterations

Most pulmonary fibrosis patients who have a background of familial clustering of familial interstitial pneumonia show mutations in genes that encode surfactant-associated protein C (*SFTPC*) [178,179], surfactant-associated protein A2 (*SFTPA2*) [180], telomerase components (*TERT* and *TERC*) [181,182], and genes associated with telomere biology, such as poly (a)-specific ribonuclease deadenylation nuclease (*PARN*) and regulator of telomere elongation helicase 1 (*RTEL1*) [183]. Further, several common variants in *TERT*, genomic regions near *TERC*, oligonucleotide-binding fold containing 1 (*OBFC1*), *RTEL1*, *PARN*, and toll-interacting protein (*TOLLIP*), have all been associated with a sporadic risk of developing IPF [184,185,186]. Nevertheless, the most important genetic risk factor for sporadic IPF is a common variant (rs35705950) in the promoter region of the *MUC5B* gene, although it is also associated with familial pulmonary fibrosis [148] (Table 4). 

LC development is the result of a stepwise accumulation of multiple acquired mutations of tumour suppressor genes or candidates, and the overexpression and mutation of oncogenes (Table 4). In this context, multiple *P53* gene mutations have been found during the early stage of bronchial carcinoma [187,188]. Frequent *P53* gene alterations have also been detected in epithelial lesions from IPF patients [189] and in squamous metaplasia, distributed in the peripheral zone of the fibrotic area in patients with IPF [190]. Similarly, aberration and loss of function of the candidate tumour suppressor gene fragile histidine triad (*FHIT*) has been reported in NSCLC [191], as well as in IPF lesions [192]. Additionally, *FHIT* gene allelic loss has been seen more frequently among the metaplasias and bronchiolar epithelia samples obtained from LC-IPF patients than from IPF patients without LC [192]. Otherwise, the frequency of expression of Ras protein in ATII cells has been observed as being significantly greater in lung tissues from LC-IPF patients compared with lung tissues from IPF patients who did not have lung carcinoma. Moreover, a specific point mutation in codon 12 of the *KRAS* gene has been detected in LC-IPF patients [189]. Interestingly, this mutation has not been identified between the numerous *KRAS* mutations observed in lung carcinoma tissue [193]. However, contrasting results regarding the prevalence of *KRAS* mutations in LC-IPF patients have been reported recently [194,195]. In addition, a significantly higher prevalence of *BRAF* mutations in IPF-LC than in LC has been observed, with an equal distribution between ADC and SQC subtypes. Moreover, some of these somatic mutations have not been shown to be significant in NSCLC patients [195]. As such, it is rational to suggest that these somatic mutations in tumour suppressor genes and oncogenes, as well as oncogene overexpression, predispose IPF patients to develop LC. However, it also raises two controversial questions: Why is LC not the cause, instead of a consequence, of IPF?; and why do LC and IPF not independently and synchronously develop as a result of common pathogenetic mechanisms? In answering these questions, Hwan et al. [195] revealed a predominance of C>T somatic transitions in most of the somatic mutations detected in LC-IPF patients. By contrast, in the non-IPF SQC subtype, C>A transversions are the most frequent [196]. This suggests a potential association between APOBEC (cytidine deaminase, which converts cytosine to uracil)-related mutagenesis and the development of LC associated with pulmonary fibrosis. 

Recently, germline mutations associated with familial NSCLC and predisposing to it are also being discovered [197] (Table 4). In this context, several findings suggest that some germline mutations that predispose patients to develop IPF also predispose them to developing lung carcinoma. Indeed, two heterozygous missense mutations, and a heterozygous missense mutation in *SFTPA2* [198] and *SFTPA1* [199], respectively, have been identified in LC-IPF families. All of these mutations are predicted to disrupt the structure of surfactant A protein (SP-A) and impair protein secretion [198,199], leading to protein instability and ER stress of resident ATII cells [179,200]. SP-A is produced by ATII and club cells [201], which have both been proposed as possible initiators of lung ADC [202]. Although the role of ER-stress in tumourigenesis is controversial [69,70], recent evidence showing that ER stress may attenuate senescence and promote tumorigenesis might explain the co-occurrence of LC (histological subtype ADC) and pulmonary fibrosis in families with an *SFTPA1/2* mutation [71]. Two further germline mutations in *TERT* (rs2736100) and *CDKN1A* (rs2395655), which were previously reported to confer IPF risk [203], have also been identified in several LC-IPF patients [195]. These mutations affect telomerase function and impair the cellular response to DNA damage, respectively [204]. Accordingly, they might also explain the co-occurrence of both diseases. Furthermore, the germline variant, rs2736100, has been reported to be associated with lung ADC risk [205], and other *TERT*, *TERC*, *OFBC1*, and *RTEL1* polymorphisms have also been revealed as risk factors of LC [206,207]. However, telomere functionality and its contribution to LC development is controversial. In fact, a gain at chromosomal region 5p15.33 in *TERT* is the most frequent genetic event in the early stages of NSCLC [208]. However, short telomeres in peripheral blood leukocytes have been related to an increased risk of lung carcinoma [209], probably via an increased mutation rate and the genomic instability induced by telomere dysfunction [210]. Therefore, it might be hypothesised that mutations associated with telomere biology in IPF lesions, which correlate with shortened telomeres in leukocytes and ATII cells [211], could drive the development of LC via an increased mutation rate and genomic instability.

Finally, the germline or somatic variant (rs35705950) in the *MUC5B* promoter region that consists of TT and GT genotypes (risk genotypes for IPF) has been reported to confer a survival advantage among patients with IPF [212]. However, these genotypes are associated with poorer overall survival in NSCLC patients [213]. Furthermore, significant associations between the *MUC5B* promoter polymorphism and the incidence of radiation pneumonitis in patients with NSCLC have not been identified [213]. This supports the idea that IPF underlies the development of LC and is not a consequence of it. 

### 5.2. Epigenetic Alterations

Due to similar pathogenic mechanisms between IPF and LC, their global methylation patterns are also somewhat similar (Table 4). However, there are also differences, which may be explained partly by IPF or cancer-specific changes [214]. For example, it was found that as a consequence of promoter hypermethylation, the relative expression of the *SMAD4* gene was significantly lower in the tumours of LC-IPF patients compared to those who had LC without IPF [215]. This was a surprising finding because *SMAD4* has been identified as a tumour-suppressor gene [216], but TGFβ1 signalling is the main effector in pulmonary fibrosis. Thus, *SMAD4* over-expression would be expected in this disease. Another epigenetic alteration involved in IPF is *THY-1* promoter hypermethylation and the absence of fibroblast Thy-1 expression, which is linked to the transformation of fibroblasts into myofibroblasts [217]. Loss of this molecule has also been documented in cancer and is associated with a more invasive disease [218]. By contrast, promoter hypermethylation of the *O*-6-methylguanine DNA methyltransferase (*MGMT*) gene is one of the early epigenetic marks in LC [219], while in IPF fibroblasts, *MGMT* is one of the most hypomethylated genes [220]. 

Otherwise, ~10% of miRNAs are abnormally expressed in IPF [95]. These variations are all capable of influencing EMT and inducing the regulation of apoptosis or ECM [95]. Some of these variations are also found in LC. For example, common to IPF, mir-21 is overexpressed in LC [95], which is an independent negative prognostic factor for overall survival in NSCLC patients [221]. By contrast, Let-7d expression is found to be mostly down-regulated in IPF and LC and acts as an oncogene [95,219,222].

## 6. Therapeutic Management in Lung Cancer Associated with Pulmonary Fibrosis Patients

The focus of IPF treatment in previous decades has been to use anti-inflammatory/immunomodulatory drugs in combination with antioxidants. However, their therapeutic usefulness was recently questioned given the unfavourable outcome when N-acetyl L-cysteine (NAC) was used in combination with prednisolone/azathioprine [224]. Following this, NAC monotherapy results were also negative [225], although a subgroup of IPF patients with the rs3750920 (*TOLLIP*) TT genotype showed a favourable response [226]. Numerous cellular and preclinical studies hold that antioxidants protect against cancer [227,228]. However, it has been shown that NAC increases tumour progression and reduces survival in LC preclinical models [229], which contraindicates NAC treatment for LC-IPF.

In line with the antioxidant treatments tested in IPF, pirfenidone was initially considered as an antioxidant therapy since it demonstrated O_2_^−^ scavenging activity [230,231]. Oral NAC has been used in conjunction with pirfenidone to treat IPF, but it does not substantially alter the tolerability profile of pirfenidone and is unlikely to be beneficial in IPF [232]. Beyond its antioxidant activity, pirfenidone is a pleiotropic molecule that inhibits TGF−β, collagen synthesis, and fibroblast proliferation, and also mediates tissue repair [233,234,235,236]. It is currently approved as an IPF therapy and Miuri et al. [237] observed that the incidence of LC in IPF patients treated with pirfenidone was significantly lower than in a non-pirfenidone IPF patient group. Furthermore, recent publications have shown that pirfenidone confers anti-fibrotic effects by interfering with the shh pathway [238], which can partly explain the observed lower LC incidence in IPF patients treated with pirfenidone. It has also been observed that perioperative pirfenidone treatment reduces the incidence of postoperative acute exacerbation of IPF in LC-IPF patients [239]. Moreover, experimental data have shown that the combination of pirfenidone and cisplatin may lead to an increase of CAF and tumour cell mortality in NSCLC preclinical models [240].

Advances in the understanding of IPF pathogenesis have resulted in further preclinical and clinical trials of drugs with antiproliferative and antifibrotic effects. For instance, tyrosine kinase inhibitors (TKIs), such as imatinib, nilotinib, gefitinib, erlotinib, nintedanib, SU5918, and SU11657, are being investigated [241,242,243,244,245,246,247]. The important role of these inhibitors in cancer treatment was previously shown [248]. Indeed, gefitinib and erlotinib are important oral treatments for NSCLC patients with mutations that activate the epidermal growth factor receptor (EGFR). In IPF, imatinib was tested in fibrotic patients, but failed to show any benefit on survival or lung function [249]. In contrast, the VEGF, FGF, and PDGF receptor inhibitor, nintedanib, has been approved for IPF treatment. Additionally, this drug is also approved for use in combination with docetaxel as an effective second-line option for patients with advanced ADC-NSCLC who have been previously treated with one course of platinum-based therapy [250]. 

Another class of antifibrotic drugs are the mTOR kinase inhibitors, including everolimus, which failed as an IPF treatment [251]. However, everolimus has shown modest beneficial effects in patients with advanced NSCLC who were previously treated with chemotherapy alone, or with chemotherapy and EGFR inhibitors [252]. It is also approved as a second-line treatment in renal and breast cancer. Currently, there are efforts towards assessing the efficacy of a new mTOR kinase inhibitor (GSK-2126458) for IPF and advanced solid tumour treatment.

In addition to the previously mentioned therapeutic strategies, a broad range of IPF therapies are currently being tested in clinical trials (Table 5). Some of these therapies target molecules and mechanisms mentioned in this review, and which are hallmarks of the progression of both diseases. These include anti-IL-13 antibodies (QAX576 and Lebrikizumab), anti-CCL2 antibodies (Carlumab and CNTO-888), anti-TGFβ1 antibodies (Fresolimumab and GC1008), anti-integrin αvβ6 antibodies (BG0011 and STX-100), integrin αvβ6 antagonist drugs (GSK3008348), LPAR1 antagonist drugs (BMS-986020), ATX-inhibiting drugs (GLPG1690), angiostatic drugs (Tetrathiomolybdate), shh pathway-inhibiting drugs (vismodegib), and galectin-3-inhibiting drugs (TD139). Only two of these drugs are being clinically developed for NSCLC patients. Fresolimumab, which was tested in combination with stereotactic ablative radiotherapy in a phase I study, and tetrathiomolybdate in combination with carboplatin and pemetrexed, which is currently being tested in a phase I study. There are also preclinical studies for NSCLC that include some of these target molecules. For example, CCR2 antagonism was demonstrated to supress CCL2-mediated viability, motility, and invasion of the NSCLC cell line, A549, in vitro [253]. Likewise, galectin-3 knockdown in NSCLC cell line-derived sphere resulted in attenuation of lung carcinogenesis by inhibiting stem-like properties [254]. In the same way, genetic deletion of *ATX* and *LPAR1* was shown to attenuate lung carcinogenesis development in animal models [132]. Moreover, vismodegib is approved for the treatment of metastatic, local, or recurrent advanced basal cell carcinoma (BCC), although it has not been tested in NSCLC. Nevertheless, blockade of shh signaling synergistically has shown to increase sensitivity to EGFR-TKIs in primary and secondary resistant NSCLC cells [255].

Given the mechanistic similarities between LC and IPF diseases, and the concurrence of predominantly NSCLC and IPF, it is rational to consider the usefulness of the large number of approved NSCLC treatments for the management of pulmonary fibrosis. For example, Nivolumab is a new immunomodulatory agent that acts as a programmed death receptor-1-blocking antibody. One case study of an ADC patient with IPF showed a beneficial and sustained response in the lung, without any sign of IPF exacerbation after Nivolumab treatment [256]. This could be explained by the higher expression of programmed cell death ligand 1 reported in cancer cells from UIP-associated SQC versus non-UIP SQC patients [257]. Other examples of feasible IPF and LC-IPF treatment candidates include vantictumab, which interferes with Wnt signalling and has undergone Phase I trials for NSCLC (preclinical studies of Wnt pathway inhibition have also been performed in pulmonary fibrosis [258,259]), and Muc1-based therapeutic strategies. Indeed, four Muc1-based Phase III trials exploring cancer treatment have been completed, one of which used a Muc1 tandem repeat peptide as an immunogen (L-BLP25) in patients with stage III unresectable NSCLC after chemoradiation [260]. 

Finally, rovalpituzumab treatment, although not tested for NSCLC, is currently approved for SCLC, and it could also have potential in the treatment of IPF and LC-IPF, since it interferes with the Notch signalling pathway. In fact, it has been observed that artesunate ameliorates lung fibrosis via inhibiting the Notch signaling pathway in a rat bleomycin model [261]. 

## 7. Conclusions

The course of IPF disease and its resulting mortality are altered by the frequent co-occurrence of LC. This review supports the view of LC as a consequence of a genetic predisposition in IPF patients and, common cellular processes and molecular pathways between both diseases. Currently, there is no consensus regarding the treatment of patients with both diseases. However, pirfenidone and nintedanib are two novel drugs approved for IPF that have potential for treating patients with fibrosis, possibly extending the survival time and lowering the incidence of LC. However, we are some distance from realising effective therapeutic approaches that are capable of stopping the disease process, where disease progression still occurs in most IPF patients despite treatment. Nevertheless, we now have a great deal of knowledge about cancer biology and its similarities with IPF. Therefore, it seems reasonable to investigate whether specific cancer drugs may exert beneficial anti-fibrotic effects that are effective to treat LC-IPF patients. Furthermore, clinical trials that prospectively investigate the efficacy of currently approved anti-fibrotic agents (or agents under study) as treatments for LC-IPF patients are sorely needed.

## Figures and Tables

**Table 1:** Prevalence of lung cancer (LC) in idiopathic pulmonary fibrosis (IPF) patients.

Study	Number of Patients with IPF	Prevalence of LC (%)	LC-IPF Male (%)	LC-IPF Median Age	LC-IPF Smokers (%)	Reference
Nagai (1992)	99	31.3%	87.1%	70.9	87.1%	[9]
Matsusitha (1995)	20	48.2%	90%	66.4	74.3%	[13]
Park (2001)	281	22.4%	97%	66.8	88.9%	[14]
Le Jeune (2007)	1064	2.7%	ND	ND	ND	[20]
Ozawa (2009)	103	20.4%	95.2%	65.5	66.7%	[15]
Lee (2012)	1685	6.8%	94.7%	68.5	92.3%	[16]
Kreuter (2014)	265	16%	ND	ND	ND	[17]
Tomasetti (2015)	181	13%	82.6%	66.9	91.3%	[18]
Yoon (2018)	1108	2.8%	61%	65	77%	[19]
Kato (2018)	632	11.1%	94.3%	66.8	100%	[8]

ND: not determined.

**Table 2:** Lung cancer (LC) histological subtypes in patients with idiopathic pulmonary fibrosis (IPF).

Study	Number of Patients with LC-IPF	Squamous Cell Carcinoma	Adenocarcinoma	Other Histological Subtypes	Reference
Kawai (1987)	8	12.5%	75%	12.5%	[24]
Nagai (1992)	31	45.2%	35.2%	19.6%	[9]
Park (2001)	63	35%	30%	35%	[14]
Kawasaki (2001)	53	46%	46%	8%	[25]
Aubry (2002)	24	67%	29%	4%	[10]
Ozawa (2009)	21	38%	29%	33%	[15]
Saito (2011)	28	67.9%	25%	7.1%	[26]
Lee (2014)	70	40%	30%	30%	[27]
Kreuter (2015)	42	36%	31%	33%	[17]
Tomasetti (2015)	23	39%	35%	26%	[18]
Khan (2015)	34	41%	38%	21%	[28]
Guyard (2017)	18	44%	33%	23%	[29]
Yoon (2018)	27	41%	26%	33%	[19]
Kato (2018)	70	30%	20%	50%	[8]

**Table 3:** Principal fibrogenic molecules and signal transduction pathways participating in lung cancer (LC) and idiopathic pulmonary fibrosis (IPF).

	IPF	LC
**Growth Factors**		
TGFβ1	Overexpressed	Overexpressed
PDGF	Overexpressed	Overexpressed
VEGF	Overexpressed	Overexpressed
FGF	Overexpressed	Overexpressed
CTGF	Overexpressed	Downregulated
**Profibrotic mediators**		
LPA	Overexpressed	Overexpressed
Galectin-3	Overexpressed	Overexpressed
**Cytokines**	Overexpressed	Overexpressed
CCL2	Overexpressed	Overexpressed
IL-13	Overexpressed	Overexpressed
**Mucins**		
Mucin 1	Overexpressed	Overexpressed
Mucin 4	Overexpressed	Overexpressed
Mucin 5B	Overexpressed	Overexpressed
**Embryological pathways**		
Wnt pathway	Overexpressed	Overexpressed
Shh pathway	Overexpressed	Overexpressed
Notch pathway	Overexpressed	Overexpressed
**Proliferation-related pathways**		
PI3K/AKT/mTOR pathway	Overexpressed	Overexpressed
**Migration-related proteins**		
Laminin	Overexpressed	Overexpressed
Fascin	Overexpressed	Overexpressed
Hsp27	Overexpressed	Overexpressed
**Oxidative stress—related molecules**		
NOX4	Overexpressed	Overexpressed
Nrf2	Downregulated	Downregulated
**Cell-cell communication—related proteins**		
Connexin 43	Downregulated	Downregulated

TGFβ1: transforming growth factor β1; PDGF: platelet derived growth factor; VEGF: vascular endothelial growth factor; FGF: fibroblast growth factor; CTGF: connective tissue growth factor; LPA: lysophosphatidic acid; CCL2: chemokine ligand 2; IL-13: interleukin 13; PI3K: phosphoinositide 3-kinase; AKT: protein kinase B; mTOR: mammalian Target of Rapamycin; Hsp27: heat shock protein 27; NOX4: NADPH oxidase 4.

**Table 4:** Mutated genes, hypermethylated genes, and non-coding RNAs with altered expression in Idiopathic pulmonary fibrosis (IPF), lung cancer (LC), and LC-IPF patients.

	IPF	LC	LC-IPF
*Mutated Genes*			
***SFTPA1***	Yes [199]	ND	Yes [199]
***SFTPA2***	Yes [180]	ND	Yes [198]
***TERT***	Yes [181,184]	Yes [206,207]	Yes [195]
***TERC***	Yes [181,184]	Yes [206,207]	ND
***PARN***	Yes [183]	ND	ND
***OBFC1***	Yes [184]	Yes [207]	ND
***RTEL1***	Yes [183]	Yes [207]	ND
***TOLLIP***	Yes [186]	ND	ND
***MUC5B***	Yes [148]	Yes [213]	ND
***P53***	Yes [189]	Yes [187,188]	Yes [190]
***FHIT***	Yes [192]	Yes [191]	Yes [192]
***KRAS***	ND	Yes [193]	Yes [194]
***BRAF***	ND	Yes [223]	Yes [195]
***CDKN1A***	Yes [203]	ND	Yes [195]
*Hypermethylated Genes*			
***SMAD4***	ND	ND	Yes [215]
***THY-1***	Yes [217]	ND *	ND
***MGMT***	No [220]	Yes [219]	ND
*Non-coding RNAs*			
**Let-7d**	Downregulated [95]	Downregulated [219]	ND
**miR-21**	Upregulated [95]	Upregulated [221]	ND

ND: Not determined. * Reported in metastatic nasopharyngeal carcinoma [218].

**Table 5:** Development status of drugs targeting molecules and processes involved in lung cancer (LC) and Idiopathic pulmonary fibrosis (IPF).

Therapy	IPF	LC
Anti-PDGFR, VEGFR, FGFR (nintedanib)	Approved	Approved in combination with docetaxel (second-line treatment) for ADC-NSCLC
Anti-fibrotic drug (pirfenidone)	Approved	Preclinical studies for NSCLC [240]
Anti-IL13	QAX576 (NCT00532233, NCT01266135: Phase II completed)	Not studied
	Lebrikizumab (NCT01872689: Phase II completed)	
Anti-CCL2	Carlumab (CNTO-888) (NCT00786201: Phase II completed	Preclinical studies for NSCLC [253]
Galectin-3 inhibition	TD139 (NCT02257177: Phase I/II completed)	Preclinical studies for NSCLC [254]
Anti-TGFβ	Fresolimumab (GC1008) (NCT00125385: Phase I completed)	Fresolimumab (GC1008) (NCT02581787: Phase I/II suspended) (NSCLC patients)
Anti-αvβ6 integrin	BG0011 (STX-100) (NCT01371305: Phase II completed)	Not studied
αvβ6 antagonist	GSK3008348 (NCT02612051: Phase I completed)	Not studied
Anti-CTGF	Pamrevlumab (FG-3019) (NCT01262001: Phase II completed)	Not studied
LPAR1 antagonist	BMS-986020 (NCT01766817: Phase II completed)	Preclinical studies [132]
Autotaxin inhibition	GLPG1690 (NCT02738801: Phase II completed)	Preclinical studies [132]
Angiostatic agent	Tetrathiomolybdate (NCT00189176: Phase I/II completed)	Tetrathiomolybdate (NCT01837329: Phase I recruiting patients) (NSCLC patients)
mTOR inhibitor	GSK-2126458 (NCT01725139: Phase I completed)	Not studied *
	Sirolimus (NCT01462006: Not applicable Phase)	
TERT gene expression induction	Nandrolone decanoate (NCT02055456: Phase I/II (unknown recruitment status))	Not studied
Shh pathway inhibitor	Vismodegib (NCT02648048: Phase Ib completed)	Preclinical studies for NSCLC [255]
Nivolumab	Not studied	Approved for NSCLC
Notch pathway inhibitor	Artesunate (preclinical studies [261])	Rovalpituzumab (approved for SCLC)
Wnt pathway inhibitor	Preclinical studies [258,259]	Vantictumab (NCT01957007: Phase I completed) (NSCLC patients)
Muc1-based therapies	Anti-KL-6 (preclinical studies [262])	Muc1 immunogen (L-BLP25 (Phase III completed [260])) (NSCLC patients)

IPF: idiopathic pulmonary fibrosis; LC: lung cancer; NSCLC: non-small cell lung cancer; SCLC: small cell lung cancer; PDGFR: platelet derived growth factor receptor; VEGFR: vascular endothelial growth factor receptor; FGFR: fibroblast growth factor receptor; IL-13: interleukin 13; CCL2: chemokine ligand 2; TGFβ: transforming growth factor β; CTGF: connective tissue growth factor; LPAR1: lysophosphatidic acid receptor type 1; mTOR: mammalian Target of Rapamycin. * (NCT02581787: Phase I/II terminated for subjects with solid advanced tumors).

## Abbreviations

IPF	Idiopathic pulmonary fibrosis
UIP	Usual interstitial pneumonia
LC	Lung cancer
LC-IPF	Lung cancer associated with idiopathic pulmonary fibrosis
NSCLC	Non-small cell-lung cancer
ADC	Adenocarcinoma
SQC	Squamous cell carcinoma
SCLC	Small cell lung cancer
ATII	Alveolar type II cells
ECM	Extracellular matrix
EMT	Epithelial to mesenchymal transition
EndMT	Endothelial to mesenchymal transition
α-SMA	α-smooth muscle actin
CAF	Cancer-associated fibroblast
ER	Endoplasmic reticulum stress
TGFβ	Transforming growth factor β
FGF	Fibroblast growth factor
VEGF	Vascular endothelial growth factor
PDGF	Platelet derived growth factor
IL-13	Interleukin-13
CCL2	Chemokine ligand 2
CCR2	Chemokine receptor 2
BALF	Bronchoalveolar lavage fluid
LPA	Lysophosphatidic acid
LPAR1	Lysophosphatidic acid receptor 1
ATX	Autotaxin
Shh	Sonic hedhehog
TKI	Tyrosine kinase inhibitor
EGFR	Epidermal growth factor
